# Potential value of serum *Aspergillus* IgG antibody detection in the diagnosis of invasive and chronic pulmonary aspergillosis in non-agranulocytic patients

**DOI:** 10.1186/s12890-020-1125-y

**Published:** 2020-04-15

**Authors:** Qihong Yu, Jingdong He, Bin Xing, Xin Li, Hongyu Qian, Hong Zhang, Meilin Xu, Haiying Peng

**Affiliations:** 1 Department of Respiratory and Critical Care Medicine, Tianjin Chest Hospital, 261, Taierzhuang South Road, Jinnan District, Tianjin, 300222 China; 2 The clinical laboratory, Tianjin Chest Hospital, Tianjin, 300222 China; 3 Department of Thoracic Surgery, Tianjin Chest Hospital, Tianjin, 300222 China; 4 Department of Medical Imaging, Tianjin Chest Hospital, Tianjin, 300222 China; 5 Department of Pathology, Tianjin Chest Hospital, Tianjin, 300222 China

**Keywords:** Pulmonary aspergillosis, Non-agranulocytic patients, Serum *Aspergillus* IgG, Diagnosis

## Abstract

**Background:**

At present, serum *Aspergillus* IgG and IgM antibody detection is mainly used in the diagnosis of chronic pulmonary aspergillosis (CPA), but its value in the diagnosis of invasive pulmonary aspergillosis (IPA) in non-agranulocytic patients is still unclear. IgM can be used as a marker of acute infection to help diagnose acute infection-related diseases. IgG is a marker of long-term infection and is used to assist in the diagnosis of pre-existing or chronic infection-related diseases. The aim of this study was to investigate and compare the value of serum *Aspergillus* IgG and IgM antibody detection in the diagnosis of IPA and CPA in non-agranulocytic patients.

**Methods:**

Fifty-eight cases of pulmonary aspergillosis (37 IPA and 21 CPA cases), 15 cases of community-acquired bacterial pneumonia and 50 cases in the healthy control group were collected. The serum (1,3)-β-D-glucan test (G test) was performed with a chromogenic method, and the galactomannan test (GM test) and *Aspergillus* IgG and IgM antibody detection were performed by commercial enzyme-linked immunosorbent assay (ELISA) in all patients. The sensitivity and specificity, cut-off value and area under the curve (AUC) of *Aspergillus* IgG and IgM antibodies were further obtained by receiver operating characteristic (ROC) curves.

**Results:**

The positive rate of the G test, *Aspergillus* IgG antibody detection and the GM test also showed notable differences among the IPA, CPA, community-acquired bacterial pneumonia and healthy groups (*P* = 0.006, *P* <  0.001 and *P* = 0.217, respectively). Only the positive rate of the GM test showed a significant difference between the IPA and CPA groups (*P* = 0.04). ROC curves indicated that *Aspergillus* IgG antibody detection had a higher specificity in the IPA group than in the CPA group (0.952). The detection of *Aspergillus* IgG antibody can preferably distinguish IPA from community-acquired bacterial pneumonia and healthy controls (sensitivity = 0.923, specificity = 0.459, cut-off value = 134.46, AUC = 0.727). It can also distinguish CPA from community-acquired bacterial pneumonia and healthy controls (sensitivity = 0.952, specificity = 0.692, cut-off value = 75.46, AUC = 0.873).

**Conclusions:**

Serum *Aspergillus* IgG antibody detection may have certain clinical value in the diagnosis of IPA and CPA in non-agranulocytic patients.

## Background

Pulmonary aspergillosis is a type of lung disease caused by *Aspergillus* infection or the inhalation of *Aspergillus* antigen. Pulmonary aspergillosis is uncommon in non-agranulocytic patients, and only a small amount of data are available. Nevertheless, in recent years, the incidence of pulmonary aspergillosis in non-granulocytic patients has increased with ageing; the increase in chronic diseases; the use of broad-spectrum antibiotics, hormones, and immunosuppressive drugs; and invasive operations [1, 2]. Moreover, the clinical manifestations of these patients lack specificity, and the diagnosis is usually difficult, which leads to treatment delay and affects the prognosis. According to the clinical characteristics, pulmonary aspergillosis can be divided into allergic bronchopulmonary aspergillosis (ABPA), chronic pulmonary aspergillosis (CPA), invasive pulmonary aspergillosis (IPA), and subacute invasive aspergillosis (SAIA) [3]. Among them, CPA usually occurs in immunocompetent individuals with underlying respiratory disorders, and the prevalence of CPA worldwide is approximately 3 million [4]. Unfortunately, respiratory physicians may not detect CPA until the disease progresses to an advanced stage owing to the lack of specific clinical manifestations. More seriously, without timely diagnosis and long-term antifungal treatment, the 5-year mortality rate of patients with CPA reaches 80% [5]. Furthermore, invasive pulmonary aspergillosis (IPA) has become a common type of severe pneumonia with the highest mortality, and one of the important reasons the is difficulty in diagnosis [6]. In addition, patients with agranulocytosis are predominant among those with IPA, and relevant international guidelines for diagnosis and treatment also focus on them [7].

The diagnosis of pulmonary aspergillosis depends on histopathology and microbiological culture, but there are risks in obtaining tissue specimens. Traditional microbiological culture has a low positive rate, takes a long time, and has the possibility of contamination and colonization. However, serological diagnosis as a non-invasive diagnostic method is conducive to the early diagnosis of pulmonary aspergillosis but avoids over-diagnosis. However, this method has a false-positive reaction during the detection process, which reduces the sensitivity. The method has the advantages of high efficiency and time savings, high specificity, and high sensitivity and is suitable for the detection of a large number of samples. IgM antibody has a short half-life and disappears quickly; therefore, it can be detected in blood as an indicator of recent infection. IgG antibodies are characterized by late production, long maintenance time, slow disappearance and high concentration. Therefore, its detection in blood can be used as an indicator of long-term infection. Among the serological diagnoses, it is well known that serum *Aspergillus* IgG and IgM antibody detection is mainly used in the clinical diagnosis of CPA [8]. Related research demonstrated that *Aspergillus fumigatus* (*A. fumigatus*)-specific IgG was elevated in 98.4% (239/243) of CPA subjects [9]. In addition, at a cut-off value of 27 mgA/L, *A. fumigatus*-specific IgG is a reliable test for the diagnosis of CPA (area under the receiver operating characteristic curve (AUROC): 0.976, sensitivity: 95.6%, specificity: 100%) [10]. Synchronously, the serum G test and GM tests are mainly used for the diagnosis of IPA in agranulocytic patients. The G test detects (1,3)-β-D-glucan, and (1,3)-β-D-glucan is a polysaccharide component of the cell wall of yeast and filamentous fungi that is most useful as a fungal antigen. The GM test was used to detect galactomannan antigen, a component of the *Aspergillus* cell wall. However, these two tests have low positive rate and poor sensitivity in non-agranulocytic patients. In this study, we explored the value of the G test, GM test, and serum *Aspergillus* IgG and IgM antibody detection for the diagnosis of IPA and CPA in non-agranulocytic patients.

## Methods

### Patients and data collection

Fifty-eight pulmonary aspergillosis cases in non-agranulocytic patients admitted to Tianjin Chest Hospital from July 2017 to July 2018 were enrolled. The diagnostic criteria referred to the consensus of experts in the diagnosis and treatment of pulmonary mycosis and the criteria of the European Organization for Research and Treatment of Cancer (EORTC) [11, 12]. The exclusion criteria were as follows: (1) agranulocytic patients, (2) patients with other lung diseases, (3) patients with possible pulmonary aspergillosis, (4) patients with allergic bronchopulmonary aspergillosis, and (5) patients who were positive for human immunodeficiency virus (HIV).

The proven IPA patients required pathological histological evidence or a positive *Aspergillus* culture in a sterile site. The diagnostic criteria of probable IPA included the following: (1) patients had risk factors for pulmonary aspergillosis (such as neutropenia, transplantation, and immunosuppressive therapy), (2) patients had certain clinical manifestations of IPA, (3) imaging results were abnormal, and (4) there was microbiological evidence of IPA.

The diagnostic criteria of CPA were as follows: (1) chronic lung symptoms (cough, expectoration, haemoptysis, weight loss) for more than 3 months, (2) progressive imaging abnormalities (new or progressive cavities, infiltration around the cavity, thickening of the pleura, fungal balls), (3) microbiological evidence of CPA (the culture of sputum, bronchoalveolar lavage fluid and bronchoscopy was positive and the blood G test and GM test were positive), and (4) no or a low degree of immune impairment.

During the same period, 15 cases of community-acquired bacterial pneumonia and 50 healthy individuals served as control groups. The sex and age of the control groups were not significantly different from those of the pulmonary aspergillosis group. The following data were collected: demographic data (age, sex, weight), serum indexes, imaging features, biochemical indicators, bacterial and fungal culture results, bronchoscopic findings, and treatment outcomes. In addition, all participants signed informed consent voluntarily, and the study was approved by the ethics committee of Tianjin Chest Hospital (protocol number: 2018KY-009-01).

### Serological testing

Five millilitres of venous blood was extracted before the administration of any antibiotics. Serum was separated from the blood for immediate testing or was stored frozen at − 80 °C for later testing.

#### G test

The serum (1,3)-β-D-glucan test (G test) was conducted with a chromogenic method using a (1–3)-β-D-glucan detection kit (Dynamiker Biotechnology Co., Ltd., Tianjin, China). In brief, a 5 μl serum sample was first pretreated for 10 min at 37 °C with 20 μl of a solution containing 0.6 M KCl and 0.125 M KOH and then assayed with Glucatell reagent in a kinetic, chromogenic format for 30 min at 37 °C. Subsequently, the optical densities at 405 nm (OD405) were read. Finally, the concentration of G in each sample was calculated by using a calibration curve with standard solutions of 6.25 to 100 pg/ml. Cases were judged positive if the level of G was ≥120 pg/ml in at least one serum sample [13].

#### GM test

The serum galactomannan test (GM test) was carried out with a commercial enzyme-linked immunosorbent assay (ELISA) kit (Dynamiker Biotechnology Co., Ltd. Tianjin, China) according to the manufacturer’s instructions. The judgement criteria for the GM test results were as follows: ≥ 0.85 μg/L was considered positive, < 0.65 μg/L was considered negative, and 0.65–0.85 μg/L was considered intermediate [14].

#### Aspergillus IgG

The commercial ELISA kit (Dynamiker Biotechnology Co., Ltd. Tianjin, China) was used to detect *Aspergillus* IgG antibody, and the experimental procedure followed the instructions. An *Aspergillus* IgG concentration ≥ 120 AU/ml was considered positive, < 80 AU/ml was considered negative, and 80–120 AU/ml was considered intermediate [15].

#### Aspergillus IgM

According to the manufacturer’s instructions, *Aspergillus* IgM antibody was detected by a commercial enzyme-linked immunosorbent assay (ELISA) kit (Dynamiker Biotechnology Co., Ltd. Tianjin, China). The judgement criteria for *Aspergillus* IgM detection included the following: ≥ 120 AU/ml was considered positive, < 80 AU/ml was considered negative, and 80–120 AU/ml was considered the intermediate [15].

### Statistical analysis

SPSS 21.0 software was used for statistical analysis. Comparisons between groups were performed by the chi-squared test. Fisher’s test results were used when the sample size was small and the theoretical number was small. The Mann-Whitney U test was used in the course of disease, age and serum indicators except lymphocyte count indicators. An independent-sample t test was used for lymphocyte count indicators. The sensitivity, specificity and optimal threshold were determined by receiver operating characteristic (ROC) curve analysis in the pROC package. The best cut-off value was the value that maximized the sum of the sensitivity and specificity in the ROC curve. This study defined a *P* value < 0.05 as a significant difference.

## Results

### Patient characteristics

The characteristics of the 58 pulmonary aspergillosis patients are shown in Table 1. There were 36 males and 22 females, aged from 46 to 75 years (60.7 ± 14.6), and 37 IPA (63.8%) and 21 CPA cases (36.2%) were included. The IPA cases included 3 proven patients and 34 probable patients, with no possible patients. The CPA cases included 3 simple aspergilloma (SA), 10 chronic cavitary pulmonary aspergillosis (CCPA) and 8 subacute invasive aspergillosis (SAIA) cases [16]. Among these pulmonary aspergillosis patients, 7 patients (12.1%) has no other underlying diseases, 26 patients (44.8%) had chronic respiratory disease, and 15 patients (25.9%) had diabetes (Table 1).

**Table 1:** Characteristics of 58 pulmonary aspergillosis cases in non-agranulocytic patients

characteristics	No. of patients (*n* = 58)	%
Gender		
Male	36	62.07
Female	22	37.93
Age		
≥ 60	32	55.17
< 60	26	44.83
Case classification		
IPA	37	63.79
CPA	21	36.21
Underlying disease		
chronic respicatory disease	26	44.83
Organ failure	1	1.72
Chronic cardiovascular disease	5	8.62
pulmonary tuberculosis	4	6.90
chronic liver disease	1	1.72
Diabetes	15	25.86
Autoimmune disease	1	1.72
Others	8	13.79
No underlying disease	7	12.07

*IPA* invasive pulmonary aspergillosis, *CPA*, chronic pulmonary aspergillosis

### Characteristics comparison between IPA and CPA cases

Clinical features between IPA and CPA cases were compared and are exhibited in Table 2, including microbiological findings, clinical symptoms, thoracic CT signs, the involved lobes of the lung, and serum indexes. It was obvious from Table 2 that the course of CPA cases was longer than that of IPA cases. Some clinical symptoms, such as fever, dyspnoea and haemoptysis, were very different between IPA and CPA cases (*P* <  0.05). Observable differences were found between the above two groups in terms of thoracic CT signs of patchy exudate shadows; air crescent sign and ground-glass opacity attenuation; the involvement of the right middle, right lower and left upper lobes of the lung; and serum indexes of LDH, albumin, PCT levels and lymphocyte count (*P* <  0.05, Table 2).

**Table 2:** Comparison of clinical features between IPA and CPA cases

Characteristics	IPA (*n* = 37)	CPA (*n* = 21)	Overall (n = 58)	*P*
Ratio of male to female patients	23:14	13:8	36:22	0.985
Age, median years	64 (54.513, 72)	60 (46.524, 66)	64 (53.753, 70.248)	0.128
Course of disease (median, day)	17 (10, 30)	75 (23.251, 187.532)	20 (13, 60)	< 0.001
Microbiological findings				
*Staphylococcus aureus*	1 (2.703)	0	1 (1.724)	1.000
*Pseudomonas aeruginosa*	1 (2.703)	1 (4.762)	2 (3.448)	1.000
*Candida albicans*	1 (2.703)	1 (4.762)	2 (3.448)	1.000
Acinstobacter baumannii	1 (2.703)	1 (4.762)	2 (3.448)	1.000
*Klebsiella pneumoniae*	2 (5.405)	0	2 (3.448)	0.530
Aspergillus	8 (21.622)	3 (14.286)	11 (18.966)	0.301
Others	2 (5.405)	1 (4.762)	3 (5.172)	1.000
Clinical symptoms, *n*(%)				
Cough	34 (91.892)	18 (85.714)	52 (89.655)	0.657
Fever (> 38 °C)	22 (59.459)	3 (14.286)	25 (43.103)	0.001
Dyspnoea	25 (67.568)	8 (38.095)	33 (56.897)	0.029
Haemoptysis	13 (35.135)	13 (61.905)	26 (44.828)	0.049
Chest Pain	8 (21.622)	2 (9.524)	10 (17.241)	0.301
Expectoration	33 (89.189)	17 (80.952)	50 (86.207)	0.443
Thoracic CT signs, *n*(%)				
Patchy exudate shadows	36 (97.297)	11 (52.381)	47 (81.034)	< 0.001
Nodules	19 (51.351)	9 (42.857)	28 (48.276)	0.534
segmental areas of consolidation	4 (10.811)	7 (33.333)	11(18.966)	0.077
Cavity	17 (45.946)	14 (66.667)	31 (53.448)	0.128
Pleural effusion	11 (29.730)	2 (9.524)	13 (22.414)	0.106
Air crescent sign	2 (5.405)	9 (42.857)	11 (18.966)	0.001
ground-glass attenuation	22 (59.459)	5 (23.810)	27 (46.552)	0.009
other	1 (2.703)	13 (61.905)	14 (24.138)	< 0.001
Involving lobes of lung, *n*(%)				
Right upper lobe	28 (75.676)	13 (61.905)	41 (70.689)	0.268
Right middle lobe	24 (64.865)	3 (14.286)	27 (46.552)	< 0.001
Right lower lobe	30 (81.081)	9 (42.857)	39 (67.241)	0.003
Left upper lobe	28 (75.676)	7 (33.333)	35 (60.345)	0.002
Left lower lobe	27 (72.973)	12 (57.143)	39 (67.241)	0.217
Serum indexs				
Erythrocyte sedimentation rate (mm/h)	46 (26.515, 62)	30 (19.525, 55)	42 (25.514, 60.543)	0.369
White blood cell count (10^9^cells/L)	8.77 (5.534, 13.495)	6.97 (5.862, 8.471)	7.96 (5.788, 12.163)	0.212
Neutrophil count (10^9^cells/L)	7.3 (3.555, 11.205)	4.31 (3.475, 6.685)	5.31 (3.495, 9.945)	0.083
Eosinophilia count (10^9^cells/L)	0.07 (0.005, 0.135)	0.1 (0.035, 0.223)	0.085 (0.012, 0.153)	0.104
Monocyte count (10^9^cells/L)	0.45 (0.325, 0.57)	0.57 (0.385, 0.695)	0.49 (0.348, 0.603)	0.113
Platelet ((10^9^cells/L))	273 (199.5385.5)	257 (209,294.5)	263.5 (208.75, 336.25)	0.257
Serum ALT level (U/L)	21.8 (15.65, 41.55)	20.2 (13.9, 30.4)	21.65 (15.425, 34.775)	0.32
Serum creatinine level (μmol/L)	67 (53.75, 78.75)	67 (60, 77.5)	67 (59, 78)	0.715
Serum albumin level (g/L)	36.2 (28.725, 41)	42.8 (39.5, 44.95)	40.4 (35.6, 43.9)	<0.001
Serum LDH level (U/L)	262 (203, 373.5)	225 (187.5, 236.5)	233.5 (198, 326.75)	0.025
Serum CRP level (mg/L)	4.09 (1.135, 11)	1.7 (0.485, 7.335)	2.85 (0.538, 8.615)	0.174
PCT (ng/ml)	0.09 (0.050, 0.215)	0.05 (0.05, 0.063)	0.05 (0.05, 0.15)	0.007
Lymphocyte count (10^9^cells/L)	1.455 ± 0.63	1.890 ± 0.577	1.685 ± 0.639	< 0.001

*IPA* invasive pulmonary aspergillosis, *CPA* chronic pulmonary aspergillosis, *LDH* lactate dehydrogenase, *CRP* C-reactive protein, *PCT* procalcitonin

### Results of serum G test, GM test, and *Aspergillus* IgG and IgM antibody detection in each group

The results of the serum G test, *Aspergillus* IgG and *Aspergillus* IgM antibody detection and the GM test are listed in Tables 3, 4 and 5 for the different groups. Primitively, positive rates of the above serum indexes were found among the pulmonary aspergillosis, community-acquired bacterial pneumonia and healthy groups, and Table 3 shows the statistical results. The positive rates of the serum G test and *Aspergillus* IgG antibody detection in the pulmonary aspergillosis group were notably higher than those in the community-acquired bacterial pneumonia and healthy groups (*P* = 0.015 and <  0.0001, respectively) and were similar between the community-acquired bacterial pneumonia group and the healthy group. Afterwards, to study whether different types of pulmonary aspergillosis could be distinguished, the pulmonary aspergillosis group was divided into IPA and CPA groups according to the disease type. Table 4 shows the comparison results among the IPA, CPA, community-acquired bacterial pneumonia and healthy groups, and Table 5 shows the comparison between the IPA and the CPA groups. In addition to the G test and *Aspergillus* IgG antibody detection, the positive rate of the GM test also showed notable differences among the IPA, CPA, community-acquired bacterial pneumonia and healthy groups (*P* = 0.022) (Table 4). Nevertheless, the G test and *Aspergillus* IgG antibody detection results were not significantly different between the IPA and CPA groups (*P* ≥ 0.5), and the positive rate of the GM test was significantly different (*P* = 0.04) (Table 5).

**Table 3:** Comparisons of serum G test, Aspergillus IgG antibody, Aspergillus IgM antibody and GM test among pulmonary aspergillosis, bacterial pneumonia and healthy groups

Group	G test positive *n* (%)	Aspergillus IgG antibody positive *n* (%)	Aspergillus IgM antibody positive *n* (%)	GM test positive *n* (%)
pulmonary aspergillosis group	14 (24.138)	33 (56.897)	14 (24.138)	12 (20.690)
bacterial pneumonia group	1 (6.667)	3 (20.00)	4 (26.667)	2 (13.333)
healthy group	3 (6.000)	8 (16.000)	9 (18.000)	4 (8.000)
P	0.015	< 0.001	0.649	0.198

*G test* (1,3) beta glucan-D test, *GM test* galactomaunan test

**Table 4:** Comparisons of serum G test, Aspergillus IgG antibody, Aspergillus IgM antibody and GM test among IPA, CPA, bacterial pneumonia and healthy groups

Group	G test positive *n* (%)	Aspergillus IgG antibody positive *n* (%)	Aspergillus IgM antibody positive *n* (%)	GM test positive *n* (%)
IPA group	12 (32.430)	18 (48.650)	6 (16.220)	11 (29.730)
CPA group	2 (9.523)	15 (71.429)	8 (38.095)	1 (4.762)
bacterial pneumonia group	1 (6.667)	3 (20.000)	4 (26.667)	2 (13.333)
healthy group	3 (6.000)	8 (16.000)	9 (18.000)	4 (8.000)
*P*	0.006	< 0.001	0.217	0.022

*G test* (1,3) beta glucan-D test, *GM test* galactomaunan test, *IPA* invasive pulmonary aspergillosis, *CPA* chronic pulmonary aspergillosis

**Table 5:** Comparisons of serum G test, Aspergillus IgG antibody, Aspergillus IgM antibody and GM test between IPA and CPA groups

Group	G test positive *n* (%)	Aspergillus IgG antibody positive *n* (%)	Aspergillus IgM antibody positive *n* (%)	GM test positive *n* (%)
IPA group	12 (32.432)	18 (48.649)	6 (16.216)	11 (29.730)
CPA group	2 (9.523)	15 (71.429)	8 (38.095)	1 (4.762)
P	0.050	0.092	0.061	0.040

*G test* (1,3) beta glucan-D test, *GM test* galactomaunan test, *IPA* invasive pulmonary aspergillosis, *CPA*, chronic pulmonary aspergillosis

### ROC curves of serum *Aspergillus* IgG antibody in different groups

**Fig. 1:**
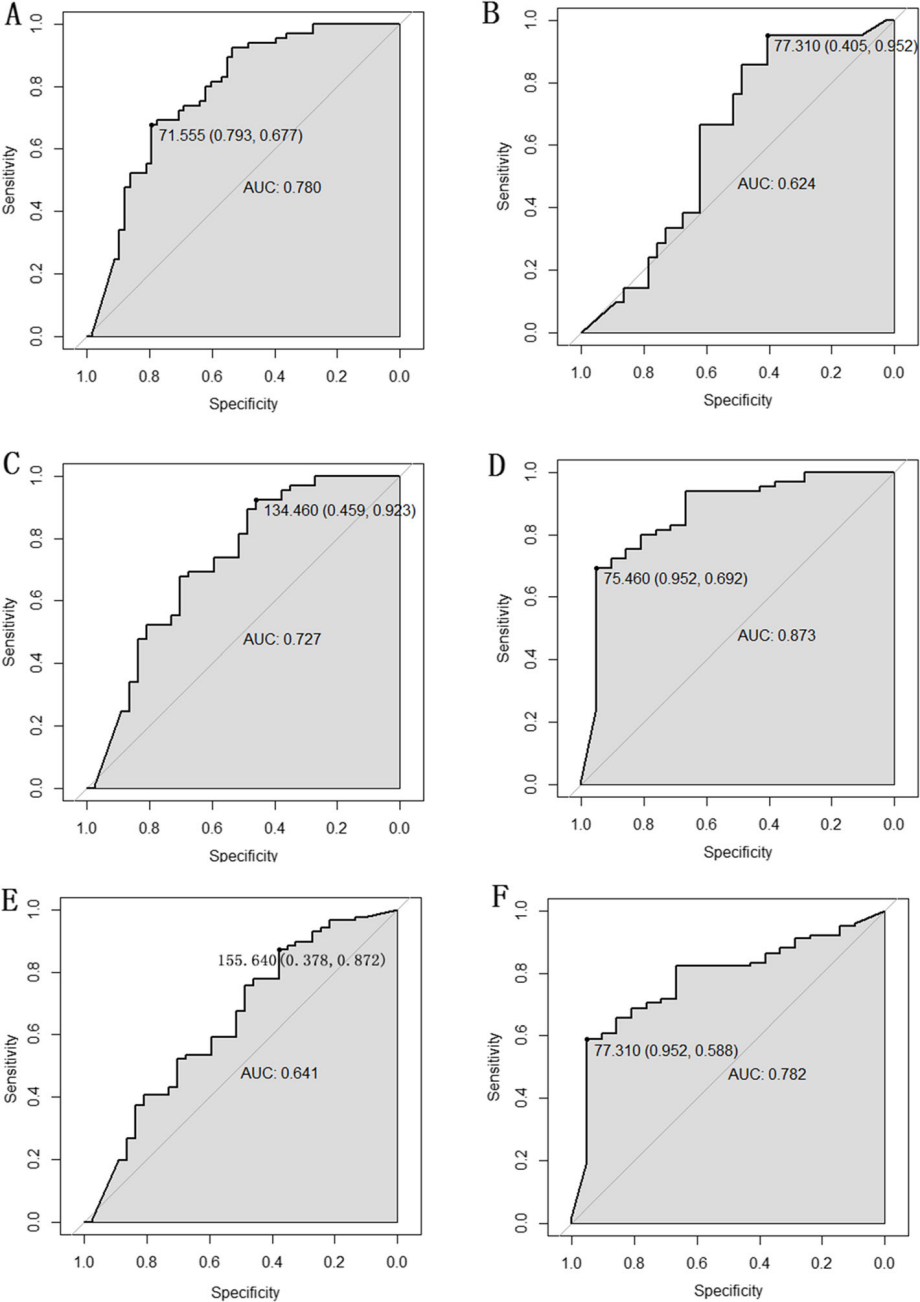
ROC curves of *Aspergillus* IgG antibody in different groups. **a**: The ROC curve of *Aspergillus* IgG antibody in the pulmonary aspergillosis group compared with the healthy group; the cut-off value (sensitivity, specificity) = 71.555 (0.793, 0.677) and AUC = 0.780, *P* < 0.001. **b**: The ROC curve of *Aspergillus* IgG antibody in the IPA group compared with the CPA group; the cut-off value (sensitivity, specificity) = 77.310 (0.405, 0.952) and AUC = 0.624, *P* < 0.001. **c**: The ROC curve of *Aspergillus* IgG antibody in the IPA group compared with the community-acquired bacterial pneumonia and healthy groups; the cut-off value (sensitivity, specificity) = 134.460 (0.459, 0.923) and AUC = 0.727, *P* < 0.001. **d**: The ROC curve of *Aspergillus* IgG antibody in the CPA group compared with the community-acquired bacterial pneumonia and healthy groups; the cut-off value (sensitivity, specificity) = 75.460 (0.952, 0.692) and AUC = 0.873, *P* < 0.001. **e**: The ROC curve of *Aspergillus* IgG antibody in the IPA group compared with the CPA, community-acquired bacterial pneumonia and healthy groups; the cut-off value (sensitivity, specificity) = 155.640 (0.378, 0.872) and AUC = 0.641, *P* = 0.013. **f**: The ROC curve of *Aspergillus* IgG antibody in the CPA group compared with the IPA, community-acquired bacterial pneumonia and healthy groups; the cut-off value (sensitivity, specificity) = 77.310 (0.952, 0.588) and AUC = 0.782, *P* < 0.001

The ROC curves of *Aspergillus* IgG antibody in different groups were drawn. Figure 1 a-f and Supplementary Table 1 display ROC curves of *Aspergillus* IgG antibody with remarkable significance (*P* < 0.05), and the cut-off value (sensitivity, specificity) and the area under the curve (AUC) are also shown. *Aspergillus* IgG antibody detection had a higher specificity (0.952) in the IPA group than in the CPA group (Fig. 1b), with the highest sensitivity (0.952) in the CPA group compared with the IPA, community-acquired bacterial pneumonia and healthy groups (Fig. 1f), and with both the highest AUC (0.873) and the highest sensitivity (0.952) in the CPA group compared with the community-acquired bacterial pneumonia and healthy groups (Fig. 1d). Furthermore, the AUC value was larger in Fig. 1D than in Fig. 1c and was larger in Fig. 1f than that in Fig. 1e. That is, serum *Aspergillus* IgG antibody detection had a better performance for distinguishing CPA than for distinguishing IPA.

## Discussion

Although pulmonary aspergillosis in non-agranulocytic patients has increased with the development of society, the frequency remains low relative to that in agranulocytic patients. To date, few data are available in non-agranulocytic patients, and most of them are found in case reports [2, 17–19]. Consequently, more cases and more studies are urgently needed to understand non-agranulocytic pulmonary aspergillosis to provide more references or clues for the diagnosis and treatment of the disease. In this article, 58 cases were reported, and the sample size was rare and large. IPA is a life-threatening infection, mainly found in patients with prolonged neutropenia. One clinical challenge of non-agranulocytic IPA cases is the frequent lack of specific clinical features, especially in patients without underlying disease [20]. In our study, we comprehensively compared clinical features between IPA and CPA cases with relevant diagnostic methods commonly used in the clinic (Table 2), including microbial cultivation, thoracic CT and serum detection. Compared with CPA, some special characteristics for IPA were spotted, such as a shorter disease course, frequent patchy exudate shadows, a specific lung lobe involvement, and lower serum albumin level, which might be used for differential diagnosis or auxiliary diagnosis.

The diagnostic gold standard of pulmonary aspergillosis mainly relies on chest imaging, microbial culture and histopathological examination. However, the imaging manifestations are poor in specificity for non-agranulocytic patients, and the phenomena of “the same disease with different image, and the different disease with same image” exist [21, 22]. For microbiological and histopathological examination, it is difficult to obtain pathological specimens, the positive rate of culture is low, and the specimens can possibly be contaminated and colonized. Therefore, the clinical diagnosis of non-agranulocytic pulmonary aspergillosis is difficult, and it is not always feasible to obtain histopathological or cytopathological demonstration of the fungus to meet the gold standard [23]. As a non-invasive diagnostic method of pulmonary mycosis, the detection of serum antigens and antibodies has attracted increasing attention. The G test and GM test are mainly used for the clinical diagnosis of IPA in agranulocytic patients, but the positive rate of IPA in non-agranulocytic patients is too low to meet clinical needs [24, 25]. For patients with agranulocytosis or severe immunosuppression, it is difficult for the body to produce an immune response. Accordingly, the detection of specific antibodies against *Aspergillus* is of little significance. With the increase in non-agranulocytic and non-immunocompromised hosts, the diagnostic significance of antibody detection for pulmonary aspergillosis needs to be re-evaluated. Serum *Aspergillus* antibody detection is mainly used for the diagnosis of CPA [5, 26]. However, the diagnostic value of *Aspergillus* antibody detection is unclear for IPA in non-agranulocytic patients because of varying results [23]. Additionally, the diagnosis of chronic pulmonary aspergillosis (CPA) is complicated, and there are limited data available [27]. Here, we compared the performances of the G test, GM test, and *Aspergillus* IgG antibody detection by using serum samples from non-agranulocytic patients with underlying pulmonary aspergillosis diseases and further subdivided IPA and CPA cases (Tables 3 and 5). The results showed that there was no significant difference in serum *Aspergillus* IgM antibodies between pulmonary aspergillosis, community-acquired bacterial pneumonia and healthy people. The reasons may include the following: 1. IgM is the earliest immunoglobulin produced after infection or immunization. It has strong bactericidal and regulatory effects, but its content in blood is low, its half-life is short, and it is susceptible to interference factors. 2. Non-granulocyte-deficient hosts may undergo a period of *Aspergillus* colonization and slow invasion before infection due to their relatively sound immune function. There are several studies about serum *Aspergillus* IgM antibody detection and its significance in the diagnosis of pulmonary aspergillosis. A multicentre prospective study evaluated the clinical performance of a commercial specific IgM antibody against *A. fumigatus* for the first time, and the results revealed that the detection of serum IgM antibody specific to *A.fumigatus* is of little help in the current diagnosis of IPA and CPA in Chinese patients, which is consistent with our results [28, 29]. IgM often occurs in the early stage of infection. Therefore, *Aspergillus* IgG antibody detection is more significant than *Aspergillus* IgM antibody detection. Our results revealed that *Aspergillus* IgG antibody reflected the greatest differences among the pulmonary aspergillosis (even IPA and CPA subdivisions), community-acquired bacterial pneumonia and healthy groups (*P* < 0.0001) (Tables 3 and 4). It was indicated that *Aspergillus* IgG antibody might be a potential diagnostic index for pulmonary aspergillosis in non-agranulocytic patients, and its performance was further evaluated through ROC curve analysis.

As exhibited in Fig. 1, *Aspergillus* IgG had notable differences in pulmonary aspergillosis (even IPA and CPA subdivision), community-acquired bacterial pneumonia and the healthy group (*P* < 0.05), and the specificity and sensitivity were 40.5–95.2% and 58.8–95.2%, respectively, and the highest AUC was 0.873. Previous studies have shown that the sensitivity and specificity of *Aspergillus* IgG antibody detection for CPA diagnosis are 75–96% and 97–99%, respectively [30]. The specificity and sensitivity were lower than those in a previous report, which might be because the underlying conditions of the research population and the experimental methods are different. Our study further certified that serum *Aspergillus* IgG antibody had better performance for distinguishing CPA than IPA. From acute invasive infection to chronic consumptive diseases, different types of pulmonary aspergillosis can overlap with each other. Generally, IPA occurs in patients with various degrees of impaired immune function, while CPA occurs in patients without or with a lower degree of impaired immune function. Therefore, serum *Aspergillus* antibody levels differ in different types of pulmonary aspergillosis, which is more significant for patients with CPA. Above all, we suspected that serum *Aspergillus* IgG detection has certain clinical value in the diagnosis of pulmonary aspergillosis in non-agranulocytic patients, especially for non-agranulocytic CPA. However, it was believed that serum *Aspergillus* IgG could not replace the traditional isolation and culture of fungi and should be combined with other diagnostic methods and clinical practice. However, this study is an exploratory study, which has the defect of insufficient supporting research literature. And some low AUC values may be caused by our small sample size. The conclusion of this study is preliminary, and we will further prove and improve it in the future research.

## Conclusions

In conclusion, serum *Aspergillus* IgG detection may have certain clinical value in the diagnosis of IPA and CPA in non-agranulocytic patients.

## Supplementary information

**Additional file 1: Table S1.** The results of ROC analysis of *Aspergillus* IgG antibody in different groups

## Data Availability

Data are available from the corresponding author upon a reasonable request.

## Abbreviations

CPA: Chronic pulmonary aspergillosis
IPA: Invasive pulmonary aspergillosis
AUC: Area under curve
APA: Acute pulmonary aspergillosis
GM test: Galactomannan test
G test: (1,3)-β-D-glucan test
ELISA: Enzyme-linked immunosorbent assay
ROC: Receiver operating characteristic curve
