# Supplementary material for: Potential value of serum Aspergillus IgG antibody detection in the diagnosis of invasive and chronic pulmonary aspergillosis in non-agranulocytic patients
Source: BMC Pulm Med. 2020 Apr 15;20:89. doi: 10.1186/s12890-020-1125-y (PMC7158007; doi:10.1186/s12890-020-1125-y)
Supplement: Supplementary file 1 — Additional file 1: Table S1. The results of ROC analysis of Aspergillus IgG antibody in different groups [file 12890_2020_1125_MOESM1_ESM.doc]

Supplementary Table 1 The results of ROC analysis of *Aspergillus* IgG antibody in different groups

| Group | Sensitivity | Specificity | Cut-off value | AUC | P-value |
| --- | --- | --- | --- | --- | --- |
| pulmonary aspergillosis group/healthy group | 0.793 | 0.677 | 71.555 | 0.780 | ＜0.001 |
| IPA/CPA | 0.405 | 0.952 | 77.310 | 0.624 | ＜0.001 |
| IPA/bacterial pneumonia and healthy group | 0.459 | 0.923 | 134.460 | 0.727 | ＜0.001 |
| CPA/bacterial pneumonia and healthy group | 0.952 | 0.692 | 75.460 | 0.873 | ＜0.001 |
| IPA/CPA, bacterial pneumonia and healthy group | 0.378 | 0.872 | 155.640 | 0.641 | 0.013 |
| CPA/IPA, bacterial pneumonia and healthy group | 0.952 | 0.588 | 77.310 | 0.782 | ＜0.001 |
